# Deflection Analysis of Layered Slabs with Plastic Inserts

**DOI:** 10.3390/ma14206050

**Published:** 2021-10-13

**Authors:** Juozas Masėnas, Remigijus Šalna, Linas Juknevičius, Juozas Valivonis

**Affiliations:** Department of Reinforced Concrete Structures and Geotechnics, Faculty of Civil Engineering, Vilnius Gediminas Technical University, Saulėtekis Ave. 11, LT-10223 Vilnius, Lithuania; remigijus.salna@vilniustech.lt (R.Š.); linas.juknevicius@vilniustech.lt (L.J.); juozas.valivonis@vilniustech.lt (J.V.)

**Keywords:** analytical calculation, layered slab, numerical model, plastic insert, shear stiffness modulus, stiffness analysis

## Abstract

The article deals with experimental and numerical research of the layered reinforced concrete slab with plastic inserts. The investigated layered reinforced concrete slab is made of prefabricated and monolithic reinforced concrete layers. Voids were formed in the plate with spherical plastic inserts. With reference to the built-up bars theory, the paper proposes an analytical method for calculating the deflection of the layered reinforced concrete structures in non-linear stage, when bond between layers is partially rigid. The article also focuses on the numerical simulation of the layered slab, compares the estimated theoretical values of deflection with the experimental values and assesses the shear stiffness of the bond of prefabricated and monolithic concrete layers for calculating the deflection of the reinforced concrete slab. Paper presents the parametric analysis of deflection dependence on shear stiffness and the width of the contact zone of the layers. It was established that proposed analytical method and numerical analysis properly characterise the behaviour of the slab. Calculation results were close to experimental data. Moreover, it was determined that performance of this type of slab is highly influenced by shear stiffness of the bond between the concrete layers. Analysis confirmed that slab fails when bond is damaged, and layers slip in the support zone.

## 1. Introduction

In construction practice, prefabricated monolithic reinforced concrete slabs are used for the installation of overlays in multi-storey buildings. A prefabricated monolithic overlay slab is a layered reinforced concrete structure consisting of prefabricated components and a layer of monolithic concrete. Filigree/Omnia prefabricated monolithic overlay slab is produced from residual reinforced concrete formwork and a layer of concrete cast in situ [1,2]. Such types of overlay slabs allow creating large hollows in the overlay. They are suitable for monolithic, prefabricated reinforced concrete, steel-framed and masonry structural systems. Such layered slabs are also easy and reliable for implementing various architectural solutions (e.g., connecting cantilever balconies to the overlay) and do not require additional formwork, which accelerates the overlay installation process. Slabs can be supported in one and both directions.

Thickness of the residual formwork (prefabricated part of the slab) ranges from 40 to 100 mm. Slab is reinforced with a reinforcing mesh that withstands tensile stress and has an anchored three-dimensional steel truss incorporated into the slab structure. Truss increases the stiffness of the residual reinforced concrete formwork and improves the bond between the prefabricated reinforced concrete slab and the monolithic concrete layer. Truss bars at the bottom act as tensile reinforcement [1,2].

**Figure 1:**
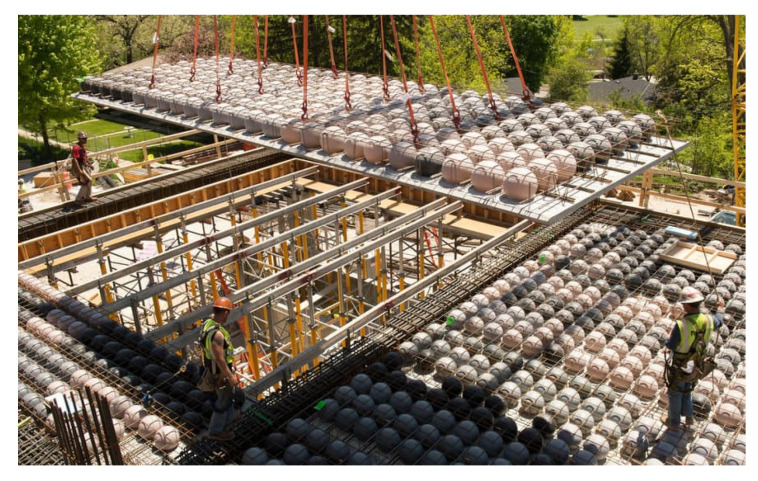
General view of residual formwork with plastic inserts [6].

To reduce the self-weight of the overlay structure, the monolithic layer of the overlay may include hollows. In this case, hollows are suggested to be formed by using plastic inserts [3,4], as shown in Figure 1. Installing hollows in the overlay slab decreases the consumption of concrete by up to 20–30%. A drop in the amount of concrete used for producing reinforced concrete overlays also diminishes the consumption of cement. 1 tonne of cement is known to emit up to 500 kg of CO_2_ into the environment [5], which demonstrates that the rational solutions of reinforced concrete structures significantly reduce energy and raw material costs and environmental pollution. Plastic inserts used for creating slab hollows can be made from household plastic waste, which grants the environmentally friendly management of plastic waste [3,4].

A number of sources [1,4,7,8,9] provide that at the initial service stage of loads affecting the above introduced structures, both layers work and deform together until loads reach 50% of bearing capacity. Conventional structural analysis applies to the previously mentioned slabs because the slab has the same load-bearing capacity as a monolithic reinforced concrete slab of the solid cross-section [3,4,10,11]. Structural analysis is possible by using conventional FEA software packages referring to the same design standards applied for the monolithic reinforced concrete slabs of the solid cross-section.

**Figure 2:**
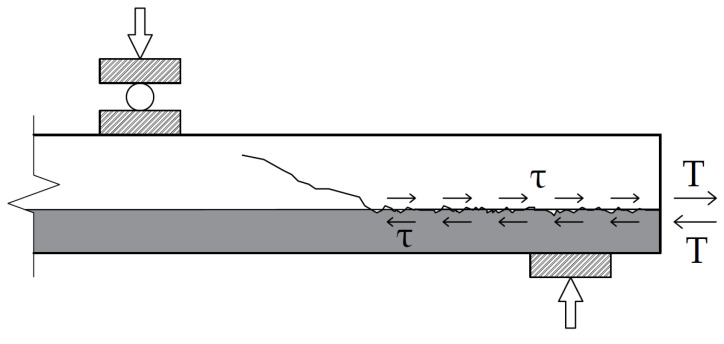
The failure mode of the layered structure in the support zone.

Structural performance of many different layered structures highly depends on the condition and properties of the bond between the layers [9,12,13,14,15,16,17,18,19,20,21,22,23]. It is caused by the shear deformations that may occur in the layer bond at the support zone of the Filigree/Omnia slab. The stiffness of the bond between two concrete layers must be considered when calculating the slabs at this particular stage of loading. At high shear stress, the rigid bond of the layers may become partially rigid, and therefore slab layers slip relatively to each other [4,24], this occurrence is shown in Figure 2. Such type of failure mode is described in studies by H. Ji and C. Liu, 2020 [25], K.M.A. Hossain et al., 2020 [26] and G. Marčiukaitis et al., 2007 [27]. In this case, the stiffness of the structure decreases and the deflection of the slab increases.

A. M. Ibrahim et al., 2019 [4] and J. Valivonis and G. Marčiukaitis 2007 [28] researched the layered reinforced concrete slabs and found that it was useful to assess the partial stiffness of the bond between concrete layers. This helps to obtain more accurate results of structural analysis and provides a possibility of assessing structural behaviour at different stages of service evaluating the partial stiffness of the bond. Furthermore, the installation of plastic hollows decreases the moment of inertia of the cross section, which in result reduces flexural stiffness of the structure. For this reason, concrete slabs with hollows usually demonstrate a larger deflection than solid slabs of same external dimensions [10,11,29,30,31].

Reinforced concrete slab with plastic inserts is a structurally, economically and ecologically advanced structural member, which is increasingly used in modern construction. Studies have been carried out on concrete slabs with plastic void formers. However, most of studies have not analysed the behaviour of semi-precast concrete slabs, which has significant advantages over a typical voided concrete slabs. This article provides findings on structural properties, concrete layer bond behaviour of layered concrete slab with plastic inserts. In addition, this paper proposes an analytical method for calculating deflection during a non-linear loading stage, when bond between layers is partially rigid.

## 2. The Analytical Method for Calculating Deflection

**Figure 3:**
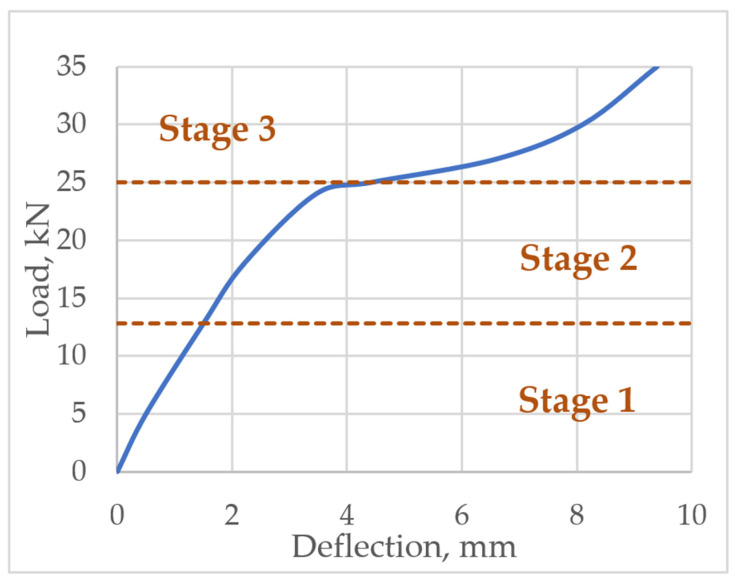
Slab loading stages.

The deflection of the layered structures is greatly influenced by the stiffness of the bond between the layers. Thus, for calculating the deflection of flexural layered reinforced concrete structures, three loading stages are identified: At stage 1, the structure works elastically and the bond between the layers is rigid; stage 2 involves plastic deformations of concrete when the member is cracked but the bond between the layers is rigid; stage 3 assumes a partially rigid bond between the layers. All stages are shown Figure 3.

At stages 1 and 2, the deflection of the layered reinforced concrete slabs is determined by applying the known methods for calculating flexural reinforced concrete structures (e.g., Eurocode 2 [32]). In this case, the two sections of reinforced concrete components are considered:

- Uncracked section, where reinforcement and concrete deform together, and full section area of concrete is considered;
- Cracked section, where the tensile zone of concrete section area is ignored.

Deflection is calculated with reference to the formula:

(1) $$w=k\cdot l_{eff}{}^{2}\cdot {\left(\frac{1}{r}\right)}_{m}\;$$

where k is the factor that assumes distribution of bending moments in the member; $l_{eff}$—the effective length (length from one support to another) of the flexural member; ${\left(\frac{1}{r}\right)}_{m}$—average curvature.

The average curvature of the flexural slab is calculated as follows:

(2) $${\left(\frac{1}{r}\right)}_{m}={\left(\frac{1}{r}\right)}_{u}\cdot \left(1-\xi \right)+{\left(\frac{1}{r}\right)}_{cr}\cdot \xi \;$$

where ξ—distribution coefficient; ${\left(\frac{1}{r}\right)}_{u}$—curvature of the uncracked cross-section; ${\left(\frac{1}{r}\right)}_{cr}$—curvature of the cracked cross-section.

The distribution coefficient can be obtained by using the following formula:

(3) $$\xi =1-\beta \cdot {\left(\frac{M_{cr}}{M}\right)}^{2}$$

where $M_{cr}$—cracking moment; M—applied bending moment; β—the coefficient which evaluates the effect of load duration and type. Under short-term load, *β* = 1.0; under long-term load, β=0.5. The research shows that the shear deformations in the layer bond of the prefabricated monolithic slab occur at the service stage when the total load acting on the structure is approximately 50% of the maximum load-bearing load. At higher shear stress, slab layers slip relatively to each other, and therefore change the stiffness of the bond between the layers. This phenomenon affects the overall flexural stiffness of the layered structure and consequently the deflection of the slab. A slip between the layers increases the deflection of the slab. Thus, for calculating prefabricated monolithic slabs at stage 3, partial rigidity between the layers must be considered. This allows determining more accurate flexural stiffness and deflection of the layered slab. The built-up bars theory [33] can be applied for estimating the deflection of the layered slab. Based on this theory, the proposed method evaluates the flexural stiffness of individual layers and the shear stiffness of the bond between the layers.

By evaluating the partial stiffness of the bond between the layers, the deflection of the two-layer prefabricated monolithic reinforced concrete slab is calculated as follows:

(4) $$w=M\cdot \left(\left(\frac{l_{eff}^{2}}{8\cdot E_{eff}\cdot I_{eff}}\right)+\frac{1}{D}\left(\frac{\cosh\left(0.5\cdot \lambda \cdot l_{eff}\right)-1}{\lambda^{2}\cdot \cosh\left(0.5\cdot \lambda \cdot l_{eff}\right)}\right)\right)$$

where M—the bending moment; $l_{eff}$—span length; $E_{eff}\cdot I_{eff}$—stiffness of the layered slab; λ—factor assessing the shear stiffness of the bond between the layers; $\frac{1}{D}$—ratio describing the flexural stiffness of the layered slab.

The ratio describing the flexural stiffness of the layered slab is calculated as follows:

(5) $$\frac{1}{D}=\frac{1}{E_{c,eff}\cdot I_{eff,1,cr}+E_{c,eff}\cdot I_{eff,2,cr}}-\frac{1}{E_{eff}\cdot I_{eff}}$$

where $E_{c,eff}$—the effective modulus of elasticity of concrete of top and bottom layers depending on creep deformations; $I_{eff,1,cr}$—effective moment of inertia of cracked section of the top layer; $I_{eff,2,cr}$—effective moment of inertia of cracked section of the bottom layer.

The factor assessing the shear stiffness of the bond between the layers:

(6) $$\lambda =\sqrt{\alpha \cdot \gamma }\;$$

where α—the factor defining shear stiffness; γ—the factor describing the flexural stiffness of the flexural layered slab.

The overall stiffness of the two-layer prefabricated monolithic reinforced concrete slab:

(7) $$E_{eff}\cdot I_{eff}=E_{c,eff}\cdot I_{eff,1,cr}+E_{c,eff}\cdot I_{eff,2,cr}+\frac{E_{c,eff}^{2}\cdot A_{eff,1,cr}\cdot A_{eff,2,cr}\cdot z_{eff}{}^{2}}{E_{c,eff}\cdot A_{eff,1,cr}+E_{c,eff}\cdot A_{eff,2,cr}}$$

where $A_{eff,1,cr}$—effective area of cracked section of the top layer; $A_{eff,2,cr}$—effective area of cracked section of the bottom layer; $z_{eff}$—the distance between the weight centres of the layers.

The factor describing the stiffness of the flexural layered slab:

(8) $$\gamma =\frac{1}{E_{c,eff}\cdot A_{eff,1,cr}}+\frac{1}{E_{c,eff}\cdot A_{reff,2,cr}}+\frac{z_{eff}{}^{2}}{E_{c,eff}\cdot I_{eff,1,cr}+E_{c,eff}\cdot I_{eff,2,cr}}$$

The factor assessing shear stiffness of the bond between the layers:

(9) $$\alpha =\frac{b\cdot G_{eff}}{z_{eff}}\;$$

where b—section width; $G_{eff}$—shear stiffness coefficient.

The effective cross-sectional area of the cracked layer:

(10) $$A_{eff,i,cr}=b\cdot x_{i,cr\;}$$

where b—section width; $x_{i,cr}$—the height of the compressive zone of the cross-section of the cracked layer; i=1,2—these numbers represent individual layers of layered slab.

The moment of inertia of the cross-section of cracked layer:

(11) $$I_{eff,i,cr}=\frac{b\cdot x_{i,cr}}{3}+\frac{E_{s}}{E_{c,eff}}\cdot A_{s,i}\cdot {\left(d_{i}-x_{i,cr}\right)}^{2}$$

where $E_{s}$—the modulus of elasticity of reinforcement steel; $A_{s,i}$—the cross-sectional area of layer reinforcement; $d_{i}$—the effective depth of the layer.

The height of the compressive zone of the cross-section of the cracked layer is calculated from first moment of area equilibrium equation. First moment of area is calculated with respect to the top of layer cross-section:

(12) $$A_{eff,i,cr}\cdot x_{cr}=S_{eff,i,cr}\to x_{cr}\;$$

where $S_{eff,i,cr}$—first moment of area of the cross-section of the cracked layer.

**Figure 4:**
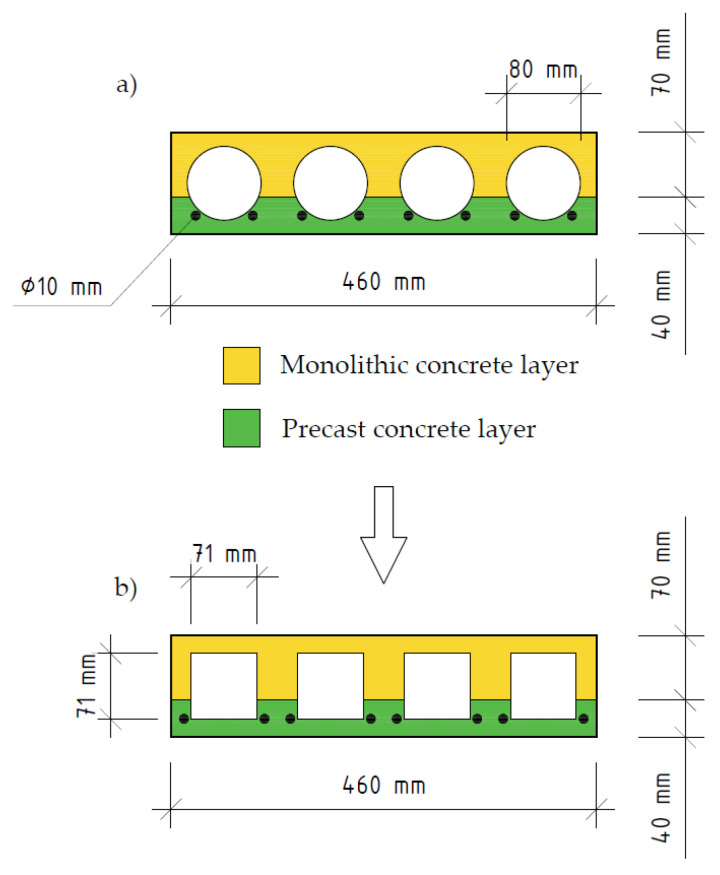
Slab cross section: (**a**) actual cross section; (**b**) transformed cross section.

In this article analytical calculations were done by transforming the cross-section with spherical voids into an I-beam type of cross-section. Both cross-sections (actual cross section, which is shown in Figure 4a, and transformed cross-section, which is shown in Figure 4b) have equal values of moment of inertia.

Usually, the effective cross section of layered slab is acquired by calculating a ratio of modulus of elasticity of individual layers. In this study this is not needed because the moduli of elasticity of both layers are equal.

## 3. Modelling of the Layered Slab

**Figure 5:**
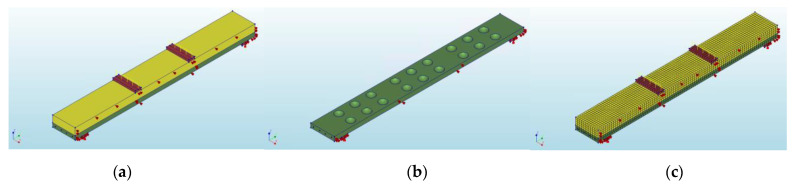
General view of the numerical model: (**a**) whole 3D slab model; (**b**) precast concrete layer; (**c**) meshed 3D model.

**Figure 6:**
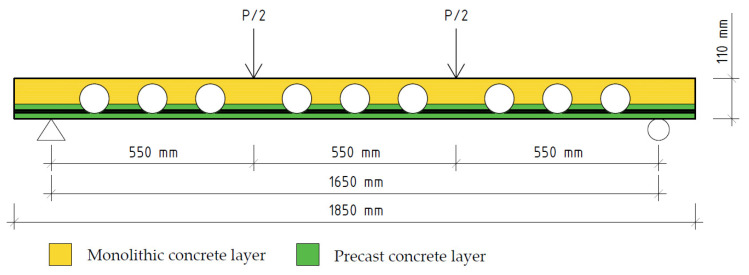
Scheme for the simulated slab.

To analyse the layered prefabricated monolithic reinforced concrete slab, a numerical model was created by using the software package of finite element analysis DIANA FEA. Numerical model can be seen in Figure 5. The geometry (which is shown in Figure 4a and Figure 6) and properties of the slab were selected with reference to the article written by A. M. Ibrahim et al., 2019 [4]. This experimental slab does not contain three-dimensional steel truss. Plastic void formers were partially embedded into bottom reinforced concrete layer at the time of its production.

**Figure 7:**
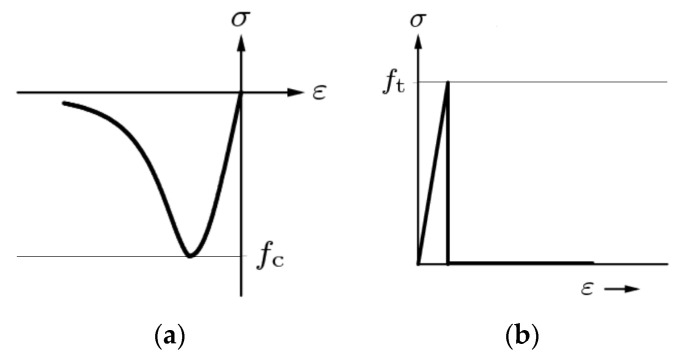
Concrete stress–strain curves: (**a**) The stress–strain relationship of compressive concrete (Thorenfeldt) [34]; (**b**) the stress–strain relationship of tensile concrete (brittle) [35].

To apply loads and supports, steel plates were simulated. Concrete in the model is treated as an elastic-plastic material. Concrete modulus of elasticity $E_{c}=32.575\;\mathrm{GPa}$; Poisson’s ratio ν=0.2; concrete density—$2400\;\mathrm{kg}/m^{3}$; stress–strain relationship curve of compressed concrete—Thorenfeldt. Curve is shown in Figure 7a; concrete compressive strength $f_{cm}=37\;\mathrm{MPa}$; stress–strain relationship curve of tensile concrete—brittle. Curve can be seen in Figure 7b; tensile strength of concrete $f_{t}=2.832\;\mathrm{MPa}$; reinforcement is treated as elastic material. Steel reinforcement modulus of elasticity $E_{s}=199\;\mathrm{GPa}$; model for steel behaviour—Von Mises plasticity, elastic stress $f_{y}=470\;\mathrm{MPa}$; reinforcement has no strengthening.

**Figure 8:**
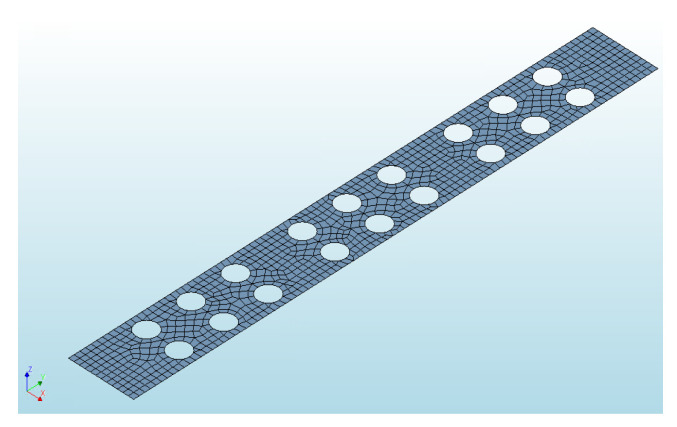
The interface surface (bond) between concrete layers.

An interface for bond evaluation is provided between concrete layers. Interface type —3D surface interface, as shown in Figure 8. Two types of interface stiffness modulus are provided. Normal stiffness modulus is equal to concrete modulus of elasticity E=33 GPa. Shear stiffness modulus is equal to G=13 GPa during stage 1. At the start of stage 3, when bond between layers becomes partially rigid and layers begin to slip, shear stiffness modulus is equal to G=2 GPa.

The finite elements of the slab are 0.02×0.02 m in size. The structure was calculated by performing nonlinear analysis and using the arch length control method [36].

## 4. The Analysis of Stress Distribution in the Numerical Model

**Figure 9:**
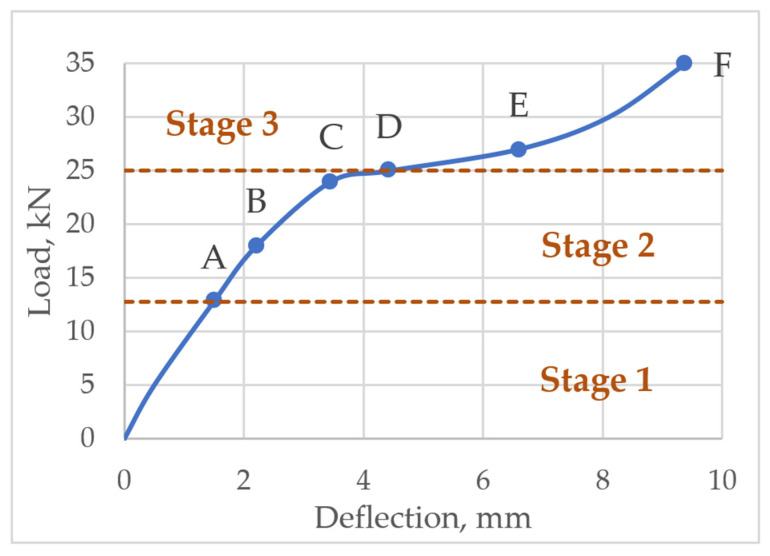
The levels of slab loading.

In order to assess the behaviour of the interface zone (bond) in the layered reinforced concrete slab, the analysis of stress distribution at the characteristic points of the cross-section was performed. The analysis was carried out at six loading levels, which are presented in Figure 9. Stress distribution in the normal section and the distribution of tangential stress in the bond between concrete layers were evaluated.

Normal stress distribution was proposed at levels B, C, D, E, F and shear stress—at levels A, C, D, E, F of an acting load. Load levels can be seen in Figure 9. Up to level A slab behaves elastically; level B and level C is at stage 2 when slab behaves plastically; at level D a slip between the layers occurs—this is the start of stage 3; at level E the layers continue to slip; at level F slab failure occurs.

**Figure 10:**
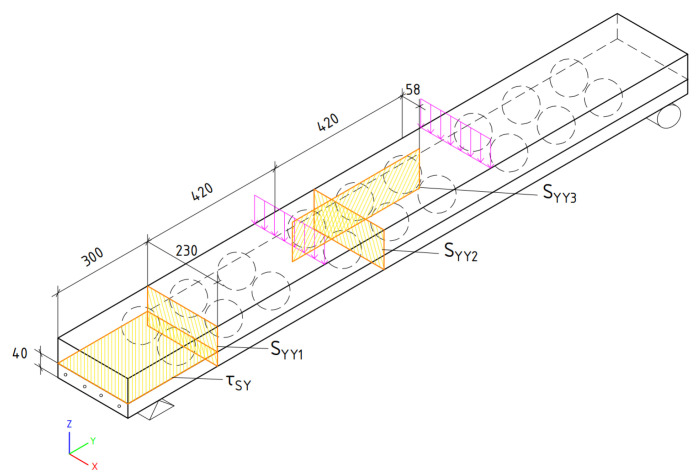
The sections for stress distribution analysis.

Normal stress is determined in three sections ($S_{YY1}$, $S_{YY2}$, $S_{YY3}$ and over slab length at the top of section $S_{YY3}$), and shear stress—in the bond between layers $\tau_{SY}$. The positions of the sections are shown in Figure 10.

**Figure 11:**
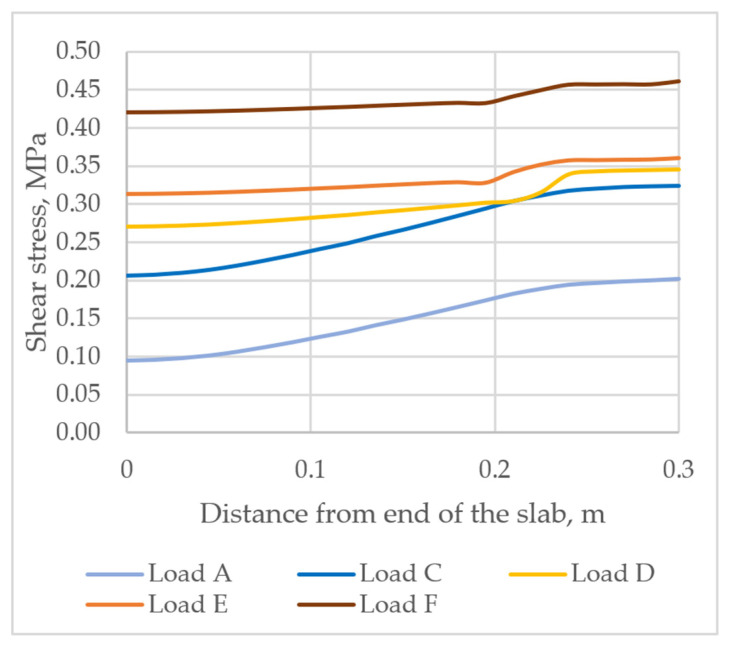
Shear stress $\tau_{SY}$ in the bond between the layers.

Under acting load A (when bond between concrete layers is not damaged), shear stress at the end of the slab is equal to τ=0.095 MPa. At a distance of 300 mm from the end of the slab, shear stress increases and reaches the value equal to τ=0.202 MPa, as shown in Figure 11. Under load C, the bond between concrete layers is still not damaged. The trend of shear stress distribution is similar to that obtained under load A. At the edge of the slab, τ=0.206 MPa, and at a distance of 300 mm, shear stress is equal to τ=0.324 MPa. Under load D, the slip between the layers occurs and a sudden rise in stress takes place at a distance of 0.225 m from the edge of the slab. τ=0.270 MPa is observed at the edge of the slab, whereas τ=0.346 MPa is determined at a distance of 300 mm from the end of the slab, as shown in Figure 11. Under load E, shear stress is distributed more evenly, and therefore spike in stress decreases. At the end of the slab, τ=0.314 MPa, whereas at a distance of 300 mm, shear stress τ=0.360 MPa. Under an increasing load (F), the layers continue to slip between each other, which can be seen in Figure 11. Shear stress distribution is similar to that under load E. At the end of the slab, shear stress τ=0.420 MPa, whereas at a distance of 300 mm, it is equal to τ=0.461 MPa. The analysis of shear distribution shows that under the acting load D (25 kN), the bond between the concrete layers of the slab becomes partially rigid, as shown in Figure 11.

**Figure 12:**
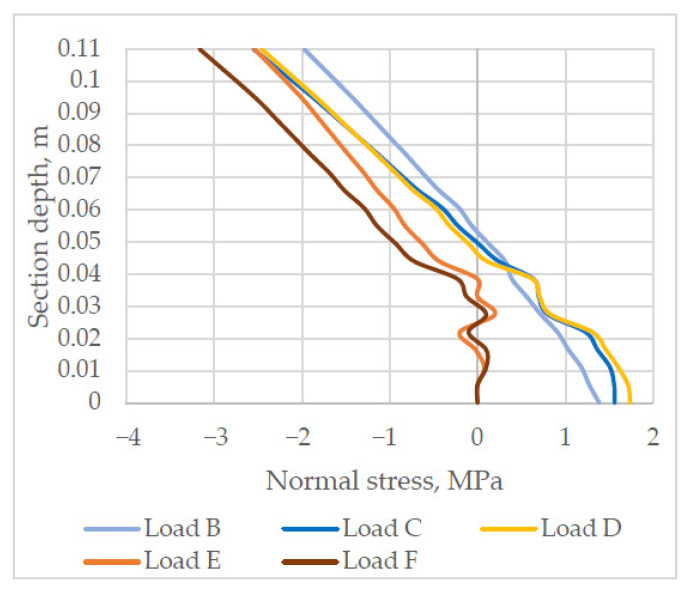
Stress distribution in the normal section of the support zone (negative stress values = compression).

The analysis of normal stress distribution in section $S_{YY1}$, which can be seen in Figure 12, shows that under load B stress is proportional to the depth of the cross-section, and in this section the layered slab behaves similarly to the solid one. Under load C, a spike is observed in the normal stress diagram at the bond of the layers. Under load D, the spike increases. Under load E, the bottom layer of the slab starts cracking. Under load F, a crack in the bottom layer further widens and compressive stress in the top layer of the slab increases.

**Figure 13:**
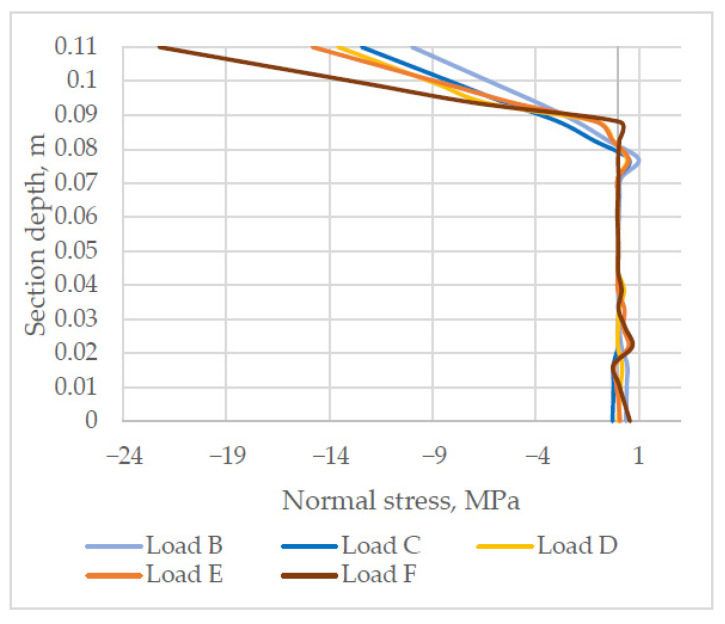
Normal stress distribution in section $S_{YY2}$ (negative stress values = compression).

The analysis of stress in section $S_{YY2}$, which can be seen in Figure 13, demonstrates that normal stress distributes proportionally over the depth of cross-section in the middle of the slab span. Yet under load B, the bottom layer and a part of the top layer of the slab crack. An increasing load causes crack development towards the top of the cross-section.

**Figure 14:**
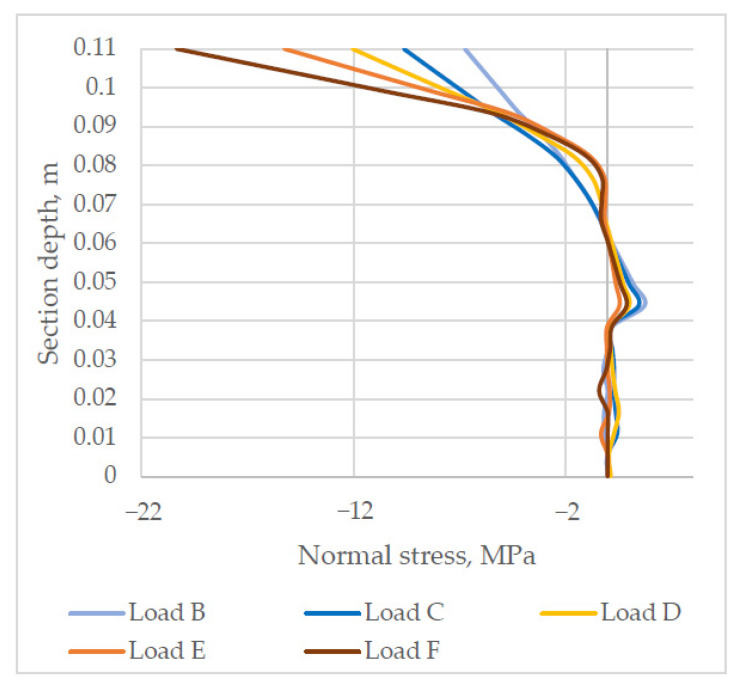
Normal stress distribution in section $S_{YY3}$ (negative stress values = compression).

In normal section $S_{YY3}$ load B causes cracks in the bottom layer of concrete. The top layer takes over all compressive stress. Tensile stress is distributed linearly, and compressive stress distribution takes the form of a parabola. Stress distribution is provided in Figure 14.

**Figure 15:**
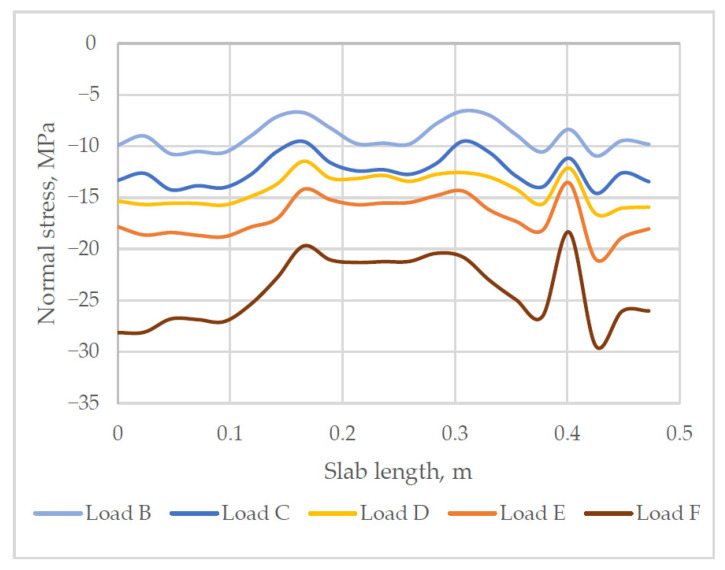
Normal stress distribution over the length of the cross-section (negative stress values = compression).

The numerical analysis has disclosed that normal cracks in the slab appear near the voids in the sections. Calculations have shown that higher stresses are concentrated near the voids and are significantly lower in concrete webs. The maximum compressive stress was observed in the areas of plastic inserts next to the load application points. Stress distribution along the length of the slab is shown in Figure 15.

## 5. The Comparison between the Analytical Calculation of Deflection and Numerical Simulation Results

The results of analytical calculations and numerical simulation are compared with the findings of experimental tests published by Ibrahim et al. [4].

The research of Ibrahim et al. determined that slab failed under load of 35 kN. Under a force of 25 kN, shear deformations in the bond of the layers are observed. An experimental slab load-deflection diagram is shown in Figure 9.

**Figure 16:**
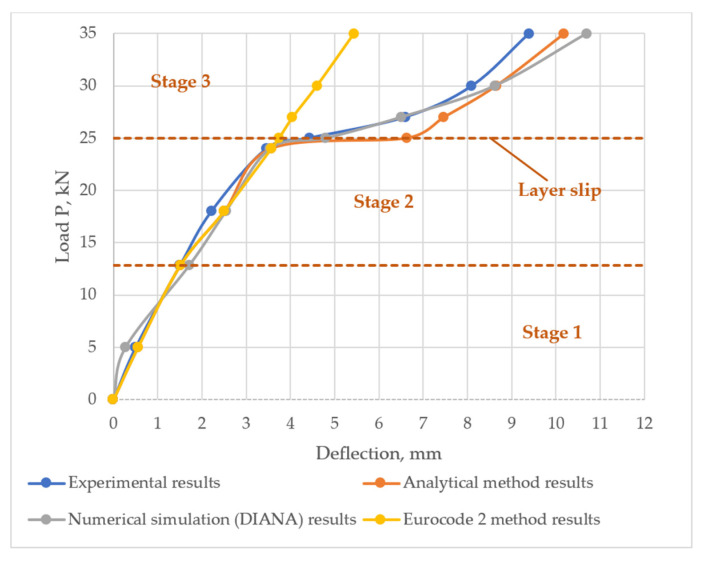
Comparison of the experimental and theoretical deflection of the prefabricated monolithic slab.

A comparison between the calculated deflections and the experimental deflection values was performed in order to validate the chosen analytical calculation method and numerical simulation results. Deflection diagrams can be seen in Figure 16. At stages 1 and 2 of the loaded structure, the calculation method given in Eurocode 2 [32] was used, and at stage 3 the method suggested by Valivonis and Marčiukaitis [28] was employed. At stages 1 and 2 of the loaded structure, analytically and numerically determined deflection values were found to be very close to experimental ones. At stage 1, in Figure 16, a linear relationship between the load and deflection values could be observed. Concrete behaves elastically and carries all the load. This type of load-deflection relationship is prevalent among all the curves in Figure 16. At stage 2, concrete starts to crack and non-linearity becomes apparent in the curves. As determined by stress distribution analysis, the bottom (precast) reinforced concrete layer is the first one to crack. Reinforcement and uncracked concrete in bottom layer carries all the load. At the stage of layer slipping (stage 3), the deflection values start to deviate from the experimental ones. When the load reaches the value of 25 kN, shear stress at supports damages the bond between the layers. Concrete layers start slipping relatively to each other. Analytical calculations determined a large increment in deflection at 25 kN load. This is because a different analytical method [28] is employed from this loading point. At this stage, the analytical deflection value is greater by 33% compared to the experimental one. From 25 kN load to the maximum load of 35 kN, concrete layers further slip. However, as deflection increases—the load increases too. This phenomenon might be caused by friction between separated layers at support zones. At the maximum load of 35 kN slab fails. Analytically obtained deflection value is greater than experimental value by 8%. Results are provided in Figure 16. The deflection values obtained by numerical analysis are close to the experimental ones at the start of layer slip. This shows that numerical model adequately assesses the properties of concrete layer bond. At 25 kN, the value is greater by 8%, whereas at the maximum load of 35 kN—by 12%. Results are presented in Figure 16.

The deflection was also calculated using the Eurocode 2 methodology. In this case it is assumed that the bond between the layers is completely rigid at all stages of loading. Load-deflection curve is linear throughout stage 1. Here concrete does not crack and behaves elastically. At stage 2, the concrete cracks. Since this methodology does not consider partial rigidity of the bond, at stage 3 the structure behaves the same as in stage 2. At the maximum load of 35 kN, the deflection value compared to the experimental one is lower by 42%, which can be seen in Figure 16.

## 6. The Parametric Analysis of Slab Deflection

**Figure 17:**
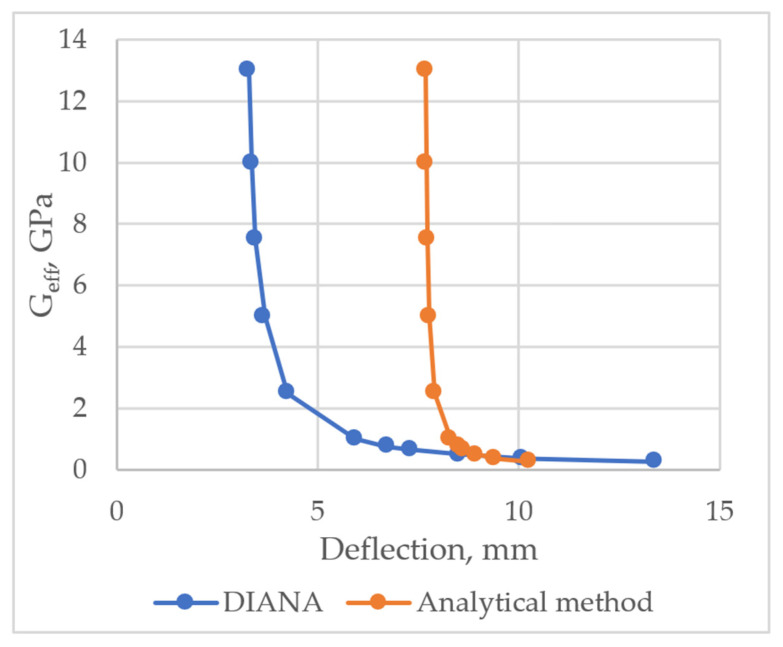
The relationship between the deflection of the prefabricated monolithic slab and the stiffness of the layer bond.

Parametric analysis was carried out by varying shear stiffness modulus $G_{eff}$. Deflection was estimated by applying DIANA FEA program and analytical methods. Deflections were calculated at P=30 kN load. When the layers of the layered structure are perfectly bonded, shear stiffness is $G_{eff}=0.4\cdot E_{c}=0.4\cdot 32.575=13\;\mathrm{GPa}$. It was found that approximately from 13 GPa to 4 GPa variations in the stiffness modulus did not have a significant effect on deflection, whereas at 4 GPa and lower, deflection started rising significantly, as shown in Figure 17.

**Figure 18:**
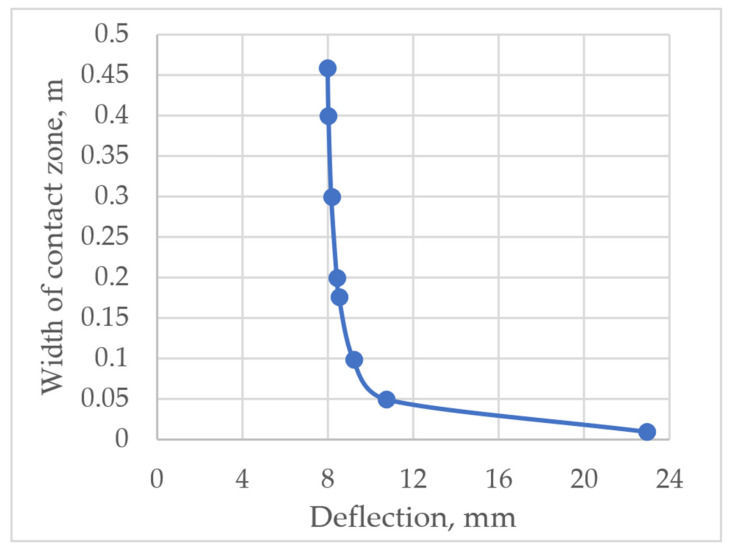
The influence of the width of the contact zone between the layers on the deflection of the prefabricated monolithic slab.

The influence of contact zone width on the deflection was also analysed. Deflection was calculated by employing the analytical method. The obtained results showed that considering the total width of the slab (approximately 0.46 m), fluctuations in the contact zone width of 0.15 m did not have a significant effect on deflection. However, starting from 0.15 m, the deflection started increasing rapidly, which can be seen in Figure 18.

## 7. Discussion

Stiffness of layered concrete slab with plastic inserts is analysed in this article. This type of concrete slab has a number of structural, economic and ecological advantages. Despite all of its positives, layered concrete slab with plastic inserts has its weaknesses. By using plastic inserts moment of inertia of the reinforced concrete slab cross section is diminished and therefore flexural stiffness of the slab is reduced. Moreover, when analysing this type of layered concrete slab, bond behaviour of two concrete layers has to be taken into consideration. Bond between concrete layers can be damaged when high shear stress near the supports occurs.

To properly assess the behaviour of layered concrete slab with plastic inserts, 3 stages of loading have been proposed. At stage 1 slab deforms elastically. Here, conventional methods for calculating flexural reinforced concrete members could be employed. At stage 2, concrete starts to crack and slab starts to behave plastically. At this stage structural member can also be analysed using typical methods for flexural reinforced concrete members. At stage 3, due to high shear stress at the support zone, the significant deformations between the layers occur, layers slip relatively to each other and thus the assessment of the partial stiffness of the bond is needed.

Layered reinforced concrete slab stiffness analysis has been conducted by employing analytical methods and numerical simulation. Acquired results were compared to experimental data from another study.

Theoretical method based on built-up bars theory was employed for analysis of layered structures. At stages 1 and 2 of the loaded structure, analytically determined deflection values were found to be very close to the experimental ones. At the stage of layer slipping (stage 3), deflection values start to differ slightly from experimental ones. Theoretical method based on built-up bars theory can be considered to be suitable for stiffness analysis of the slab.

Numerical model of layered concrete slab with plastic inserts has been built in FEA software DIANA. At stages 1 and 2, deflection results of numerical simulation were particularly close to experimental deflection values. At stage 3 numerical values start to differ slightly from ones determined in experimental study. By comparing the results, it can be stated that numerical simulation is an eligible method for stiffness analysis of layered reinforced concrete slabs with plastic inserts.

Numerical stress distribution analysis shows that when shear stress value exceeds the strength of the layer bond in the support zone, layers slip relatively to each other. When analysing normal stress distribution, it becomes apparent that bottom reinforced concrete layer is the first one to crack. Calculations have shown that higher stresses are concentrated near the voids and the loading points and are lower in concrete webs.

Parametric analysis of the slab has shown that shear stiffness modulus and the width of contact zone between the layers have non-linear influence on the deflection of layered flexural members.

Relatively small amount of available experimental data might be a limitation of this study. More experimental tests of layered concrete slabs with plastic inserts and tests of layer bond stiffness should be performed in order to completely validate the proposed analytical method.

## Data Availability

Data sharing is not applicable to this article.

## Nomenclature

w	Slab deflection	$I_{eff,1,cr}$	Effective moment of inertia of cracked section of the top layer
k	Factor, which assumes distribution of bending moments	$I_{eff,2,cr}$	Effective moment of inertia of cracked section of the bottom layer
$l_{eff}$	Effective length	α	Factor defining shear stiffness
${\left(\frac{1}{r}\right)}_{m}$	Average curvature	γ	Factor describing the flexural stiffness
ξ	Distribution coefficient	$A_{eff,1,cr}$	Effective area of cracked section of the top layer
${\left(\frac{1}{r}\right)}_{u}$	Curvature of the uncracked cross-section	$A_{eff,2,cr}$	Effective area of cracked section of the bottom layer
${\left(\frac{1}{r}\right)}_{cr}$	Curvature of the cracked cross-section	$z_{eff}$	Distance between the weight centres of the layers
$M_{cr}$	Cracking moment	b	Section width
M	Applied bending moment	$G_{eff}$	Shear stiffness coefficient
β	Coefficient, which evaluates the effect of load duration and type	$x_{i,cr}$	Height of the compressive zone of the cross-section of the cracked layer
$E_{eff}\cdot I_{eff}$	Stiffness of the layered slab	$E_{s}$	Modulus of elasticity of reinforcement steel
λ	Factor assessing the shear stiffness of the bond	$A_{s,i}$	Cross-sectional area of layer reinforcement
$\frac{1}{D}$	Ratio describing the flexural stiffness of the layered slab	$d_{i}$	Effective depth of the layer
$E_{c,eff}$	Effective modulus of elasticity of concrete	$S_{eff,i,cr}$	First moment of area of the cross-section of the cracked layer
